# Risk of HIV transmission by healthcare workers – a systematic review

**DOI:** 10.3205/dgkh000641

**Published:** 2026-03-13

**Authors:** Roland Diel, Rene Gottschalk, Albert Nienhaus

**Affiliations:** 1 Institute of Epidemiology, University Medical Hospital Schleswig-Holstein, Kiel, Germany; 2 Institute of Medical Virology, University Hospital of the Goethe University Frankfurt, Frankfurt, Germany; 3 Institution for Statutory Accident Insurance and Prevention in the Health and Welfare Services (BGW), Hamburg, Germany; 4 Competence Center for Epidemiology and Health Services Research for Healthcare Professionals (CVcare), Institute for Health Services Research in Dermatology and Nursing (IVDP), University Medical Center Hamburg-Eppendorf (UKE), Hamburg, Germany

**Keywords:** health care workers, HIV, transmission, infectivity, professional-to-patient, guidelines, exposure prone procedures, look-back studies

## Abstract

**Background:**

Occupational HIV infection among healthcare workers (HCWs) has declined markedly in high-income countries as a result of improved infection prevention and control, safety-engineered devices, and the widespread availability of antiretroviral post-exposure prophylaxis. However, the risk of HIV transmission from infected HCWs to patients remains incompletely defined.

**Methods:**

We conducted a systematic review of published studies reporting either documented HIV transmission events or the absence of transmission (look-back investigations) from HIV-infected HCWs to patients. In accordance with PRISMA guidelines, MEDLINE and Google Scholar were searched for relevant publications up to September 2025. For look-back studies with zero observed transmissions, exact binomial CIs were calculated. Pre-specified sensitivity analyses were performed to quantify statistical uncertainty arising from incomplete follow-up.

**Results:**

Of 222 records identified, 23 studies from five countries met the inclusion criteria. Direct provider-to-patient HIV transmission was documented from four source HCWs, three of whom performed exposure-prone procedures, resulting in a total of eight infected patients. The Florida dental cluster accounted for five molecularly confirmed transmissions among 1,100 tested exposed patients, corresponding to an estimated transmission probability of 0.45% (95% exact Clopper-Pearson confidence interval [CI], 0.15–1.06%), equivalent to approximately one transmission per 676 to 95 treated patients. Another single-case transmission investigation reported a lower estimated risk, with an upper confidence bound below 0.6% (approximately one transmission per 177 procedures). Two additional probable transmission events were reported without systematic testing of other exposed patients.

Sixteen look-back studies comprising 32,899 potentially exposed patients, of whom 12,924 (39.3%) underwent HIV testing, identified no HCW-to-patient transmission. In the main analyses, two-sided exact 95% CIs yielded upper transmission risk estimates ranging from 0.16% to 8.60%, largely driven by the number of patients tested. To address incomplete follow-up and major methodological biases, sensitivity analyses assuming one undetected transmission among all exposed patients were performed. Under these deliberately conservative assumptions, upper confidence limits ranged from 0.07% to 6.17%, with the majority of studies remaining below 0.5%, corresponding to fewer than one transmission per 200 exposed patients.

**Conclusions:**

With the exception of a very small number of documented provider-to-patient transmission events, the available evidence indicates that even under pessimistic assumptions the maximum plausible risk of HCW-to-patient HIV transmission remains very low. These findings support the development of clear, evidence-based national guidelines for the management of HCWs living with HIV.

## Zusammenfassung

**Hintergrund:**

Beruflich erworbene HIV-Infektionen bei medizinischem Personal (MP) haben in Hochlohnländern in den vergangenen Jahrzehnten deutlich abgenommen. Zu dieser Entwicklung haben verbesserte Maßnahmen der Infektionsprävention und -kontrolle, sicherheitsoptimierte Instrumente sowie die breite Verfügbarkeit der antiretroviralen Postexpositionsprophylaxe beigetragen. Das Risiko einer HIV-Übertragung vom MP auf Patientinnen und Patienten ist hingegen weiterhin nur unvollständig charakterisiert.

**Methoden:**

Wir führten eine systematische Übersichtsarbeit zu publizierten Studien durch, die entweder dokumentierte HIV-Übertragungen oder das Ausbleiben von Übertragungen („Look-back“-Untersuchungen) vom HIV-infiziertem MP auf Patienten berichteten. Entsprechend den PRISMA-Leitlinien wurden MEDLINE und Google Scholar nach relevanten Publikationen bis September 2025 durchsucht. Für Look-back-Studien ohne beobachtete Übertragungen wurden exakte binomiale Konfidenzintervalle berechnet. Zusätzlich wurden vorab definierte Sensitivitätsanalysen durchgeführt, um die statistische Unsicherheit infolge unvollständiger Nachverfolgung zu quantifizieren.

**Ergebnisse:**

Von 222 identifizierten Publikationen erfüllten 23 Studien aus fünf Ländern die Einschlusskriterien. Direkte HIV-Übertragungen vom medizinischen Personal auf Patienten wurden von vier Indexpersonen beschrieben, von denen drei expositionsträchtige Eingriffe durchführten. Insgesamt waren acht Patienten betroffen. Der bekannte Ausbruch in einer zahnärztlichen Praxis in Florida umfasste fünf molekularbiologisch bestätigte Übertragungen unter 1.100 getesteten exponierten Personen, entsprechend einer geschätzten Übertragungswahrscheinlichkeit von 0,45% (exaktes zweiseitiges 95%-Clopper-Pearson-Konfidenzintervall [KI] 0,15–1,06%), was etwa einer Übertragung pro 676 bis 95 behandelten Personen entspricht. Eine Einzeluntersuchung mit einem wahrscheinlichen Übertragungsereignis ergab eine niedrigere Risikoschätzung mit einer oberen Konfidenzgrenze unter 0,6% (etwa eine Übertragung pro 177 Eingriffe). Zwei weitere wahrscheinliche Übertragungen wurden berichtet, ohne dass eine systematische Testung weiterer exponierter Personen erfolgte.

Sechzehn Look-back-Studien mit insgesamt 32.899 potenziell exponierten Personen, von denen 12.924 (39,3%) getestet wurden, identifizierten keine HIV-Übertragung von MP auf Patienten. In den Haupt-Analysen ergaben die zweiseitigen exakten 95%-KI obere Risikoschätzungen zwischen 0,16% und 8,60%, wobei diese vorwiegend von der Anzahl getesteter Personen abhingen. Zur Berücksichtigung unvollständiger Nachverfolgung und größerer methodischer Verzerrungen wurden Sensitivitäts-Analysen durchgeführt, in denen jeweils eine unentdeckte HIV-Übertragung unter allen exponierten Personen angenommen wurde. Unter diesen bewusst konservativen Annahmen lagen die oberen Konfidenzgrenzen zwischen 0,07% und 6,17%; in der Mehrzahl der Studien blieben sie unter 0,5%, entsprechend weniger als einer Übertragung pro 200 exponierte Personen.

**Schlussfolgerungen:**

Abgesehen von einer sehr kleinen Zahl dokumentierter direkter Übertragungen zeigen die Daten, dass selbst unter bewusst pessimistischen Annahmen das maximal plausible Risiko einer HIV-Übertragung von MP auf Patientinnen und Patienten sehr niedrig ist. Diese Ergebnisse liefern eine robuste empirische Grundlage für die Entwicklung klarer, evidenzbasierter nationaler Leitlinien zum Umgang mit HIV-infiziertem medizinischem Personal.

## Introduction

Human immunodeficiency virus (HIV) is a blood-borne pathogen transmitted primarily through unprotected sexual contact, exposure to infected blood, perinatal transmission, and, less frequently, through occupational exposures such as needlestick injuries. It remains a major global health concern, leading to progressive immune system failure and acquired immunodeficiency syndrome (AIDS) in the absence of treatment. Although antiretroviral therapy (ART) has transformed HIV into a manageable chronic condition and significantly reduced mortality, the virus continues to cause substantial morbidity and death worldwide. According to the World Health Organization (WHO) and UNAIDS, by the end of 2024, an estimated 40.8 million people were living with HIV globally. Approximately 1.3 million people acquired HIV in that year, and about 630,000 died from AIDS-related illnesses. Critically, there is still no cure for HIV, making prevention efforts—including testing, access to ART, harm reduction, and rigorous infection prevention and control (IPC) in healthcare settings—paramount [1].

Occupational acquisition of HIV by HCWs following exposure to infected patients is well-documented [2]. However, in high-income settings, its incidence has declined markedly over the past two decades. Data from German accident insurance providers illustrate this sustained decline. The Institution for Statutory Accident Insurance and Prevention in the Health and Welfare Services (BGW) reported a reduction from 34 confirmed cases of occupationally acquired HIV between 1996 and 2017 [3] to four new cases between 2018 and 2023 [4], representing a 64.7% decrease when adjusting for the different length of the observation periods.

Comparable trends are observed internationally. In the United Kingdom, national surveillance (“Eye of the Needle”) has documented only a handful of HIV seroconversions in recent decades, with the last confirmed case following an occupational exposure reported in 1999 [5]. In the United States, the Centers for Disease Control and Prevention (CDC) documented 58 confirmed cases of occupationally acquired HIV in HCWs between the early 1980s and 2013 [6]. Notably, only one confirmed case has been reported since 1999, underscoring the rarity of such events in recent years. Together, these findings indicate that patient-to-provider transmission of HIV has become exceedingly rare in modern healthcare systems.

Against this backdrop of declining occupational acquisition, the reverse route of transmission—from an HIV-infected healthcare provider to their patients—warrants critical attention. In contrast to hepatitis B virus (HBV), such cases have been reported only rarely. The cluster of HIV infections associated with a Florida dentist in the early 1990s represents the first globally confirmed instance of HCW-to-patient transmission, where molecular epidemiology established a direct link [7], [8]. Since then, very few similar cases have been documented. Concurrently, starting with Armstrong’s 1987 study of a general surgeon who developed AIDS [9], a growing number of “look-back” studies have been published, investigating thousands of patients treated by HIV-infected HCWs and consistently finding no evidence of transmission.

Surprisingly, despite the topic's significant clinical and ethical implications, no systematic review has comprehensively synthesized the evidence on HIV transmission from infected HCWs to patients. Furthermore, the role of look-back studies—which provide crucial evidence for the absence of transmission and inform policies on practice restrictions for HIV-positive HCWs—has not been systematically evaluated.

This review therefore aims to critically appraise the published literature on HCW-to-patient HIV transmission and to assess how reported cases, or the lack thereof, have already influenced or could influence the development of international guidelines.

## Methods

### Definition of HCWs

HCWs were defined as all medical, dental, nursing, obstetric or assisting personnel working in different areas, e.g. hospitals, outpatient clinics, doctors’ practices, dialysis facilities, nursing homes and out-patient care facilities. The decisive factor was the existence of a plausible transmission pathway within these activities.

### Literature search and study selection

A comprehensive literature search up to September 30, 2025, was conducted in PubMed and Google Scholar to identify reports of HIV transmission from HCWs to patients and of look-back studies defined as retrospective investigations in which patients previously treated by an infected HCW are traced and tested to assess possible occupational transmission. Search strategies were adapted to the indexing systems and functionalities of each database to maximise both sensitivity and precision. Full search strings for each database, together with the methodological rationale for their design, are presented in Attachment 1 . Only English-language publications providing original serological data on suspected nosocomial HIV transmission to patients were considered, without restriction by publication date.

Review articles, guidelines, conference abstracts, newspaper articles, press releases, commentaries, editorials, studies without a defined HIV source and articles with a central theme diverging from or not related to reported professional-to-patient transmission of HIV were excluded. No restrictions were applied regarding study design, patient subpopulation, or mode of data collection (prospective or retrospective). If studies reported preliminary findings, the most complete and up-to-date version of the data was used. Reference lists of the included articles as well as of the review articles were manually screened to identify additional eligible publications and to remove duplicates. The Preferred Reporting Items for Systematic Reviews and Meta-Analyses (PRISMA) 2020 guidelines were followed [10], [11].

### Data extraction

Relevant studies were independently selected by two reviewing authors (RD and AN), who screened each article title and abstract initially, and then went on to review an article’s full text as required. Any discrepancies were resolved by consensus. For transmission studies, the following variables were recorded where available:

1. country and year of publication;
2. study period;
3. study design;
4. occupation or workplace of the suspected source healthcare worker (HCW);
5. number of persons tested post-exposure (excluding staff members and any secondary cases identified);
6. performance of exposure-prone procedures (EPPs);
7. HIV-1 group and subtype, if available;
8. number of transmissions, classified as confirmed, probable, or possible; and
9. suspected route of transmission.

For look-back studies, only variables 1 through 6 were assessed in the absence of documented transmissions; additionally, the total number of potentially exposed patients was recorded to allow estimation of upper confidence bounds and sensitivity analyses in the context of incomplete follow-up.

Exposure-prone procedures (EPPs) were defined as invasive procedures in which the HCW’s gloved hands or fingers may be in contact with sharp instruments, needle tips, or sharp tissues (e.g. bone or dental spicules) within a patient’s open body cavity, wound, or confined anatomical space, where the hands or fingertips may not be fully visible at all times, creating a significant risk of injury and subsequent contact with the patient’s open tissues [12].

We deliberately refrained from conducting a formal meta-analysis and summarised the findings descriptively, as the primary aim of this review was to provide a systematic overview and appraisal of the available evidence.

### Definition of transmission probability

Currently, there are no universally accepted definitions for classifying HCW-to-patient HIV transmission. For this review, a uniform classification framework was applied to ensure consistency across studies. In all cases, a minimum epidemiological prerequisite was required, including documented exposure during healthcare procedures, an appropriate temporal association, and reasonable exclusion of alternative sources of infection.

Based on this prerequisite, the level of certainty was determined by molecular evidence:

- Confirmed transmission: At least near-complete genetic identity between HCW and patient viral sequences, demonstrated by full-length or high-resolution sub-genomic sequencing (e.g., *env, gag, pol*) and/or strong phylogenetic clustering with bootstrap or posterior probability support ≥95%.
- Probable transmission: High but not definitive genetic relatedness between HCW and patient viruses, typically characterized by clustering within the same phylogenetic branch (not necessarily to the exclusion of all others) or sequence homology of generally ≥90–95% in key genomic regions, without confirmation by more detailed analyses such as quasi species comparison or transmission bottleneck assessment.
- Possible transmission: Epidemiological evidence compatible with transmission in the absence of molecular data, or with only low-to-moderate genetic relatedness in sequence comparisons. Studies reporting identical HIV subtypes alone (e.g., subtype B) or limited genotyping without higher-resolution sequencing were classified as possible transmissions unless stronger molecular evidence was provided.

### Statistics

For each transmission study reporting the number of tested exposed patients, we estimated the HIV transmission probability as the binomial proportion of confirmed patient infections among individuals in whom post-exposure serostatus was ascertained (risk=cases/tested). Two-sided 95% CIs were calculated using the exact Clopper-Pearson method, which is appropriate for rare events and zero counts.

For look-back studies in which not all potentially exposed patients were tested, pre-specified sensitivity analyses, defined *a priori*, were performed to address the resulting uncertainty and to avoid overinterpretation of zero-event findings. Two scenarios were modelled, each assuming the hypothetical occurrence of a single unrecognized HIV infection, representing the smallest non-zero event count that could plausibly have been missed. Two alternative denominator strategies were applied to capture different interpretations of uncertainty: one assuming that the missed transmission could have occurred anywhere within the entire exposed cohort (Scenario A), and a second restricting the hypothetical event to untested exposed individuals only (Scenario B).

In each scenario, the transmission probability was recalculated by assuming one hypothetical undetected HIV transmission and dividing this by either the total number of exposed patients (Scenario A) or the number of untested exposed patients (Scenario B), with two-sided 95% Clopper-Pearson CIs derived under a binomial model. These analyses were not intended to estimate the true transmission probability, but to define conservative upper bounds on the risk compatible with the available data under deliberately pessimistic assumptions.

To aid interpretation, the upper limit of the 95% CI was additionally expressed as a reciprocal “1 in x” risk, calculated as the inverse of the upper-bound probability. If studies reported molecular or epidemiological adjudication clearly excluding infections as unrelated to the suspected exposure, such cases were not counted as transmission events.

### Assessment of study quality

All studies included under these criteria were retrospective observational investigations or case reports designed either to identify the source of HIV infection or to detect secondary cases. Therefore, a formal quality assessment using standardized appraisal tools, such as the Joanna Briggs Institute critical appraisal checklist for prevalence studies, was not considered appropriate. Instead, sources of potentially relevant bias were evaluated and discussed on a case-by-case basis.

## Results

### Study availability

**Figure 1:**
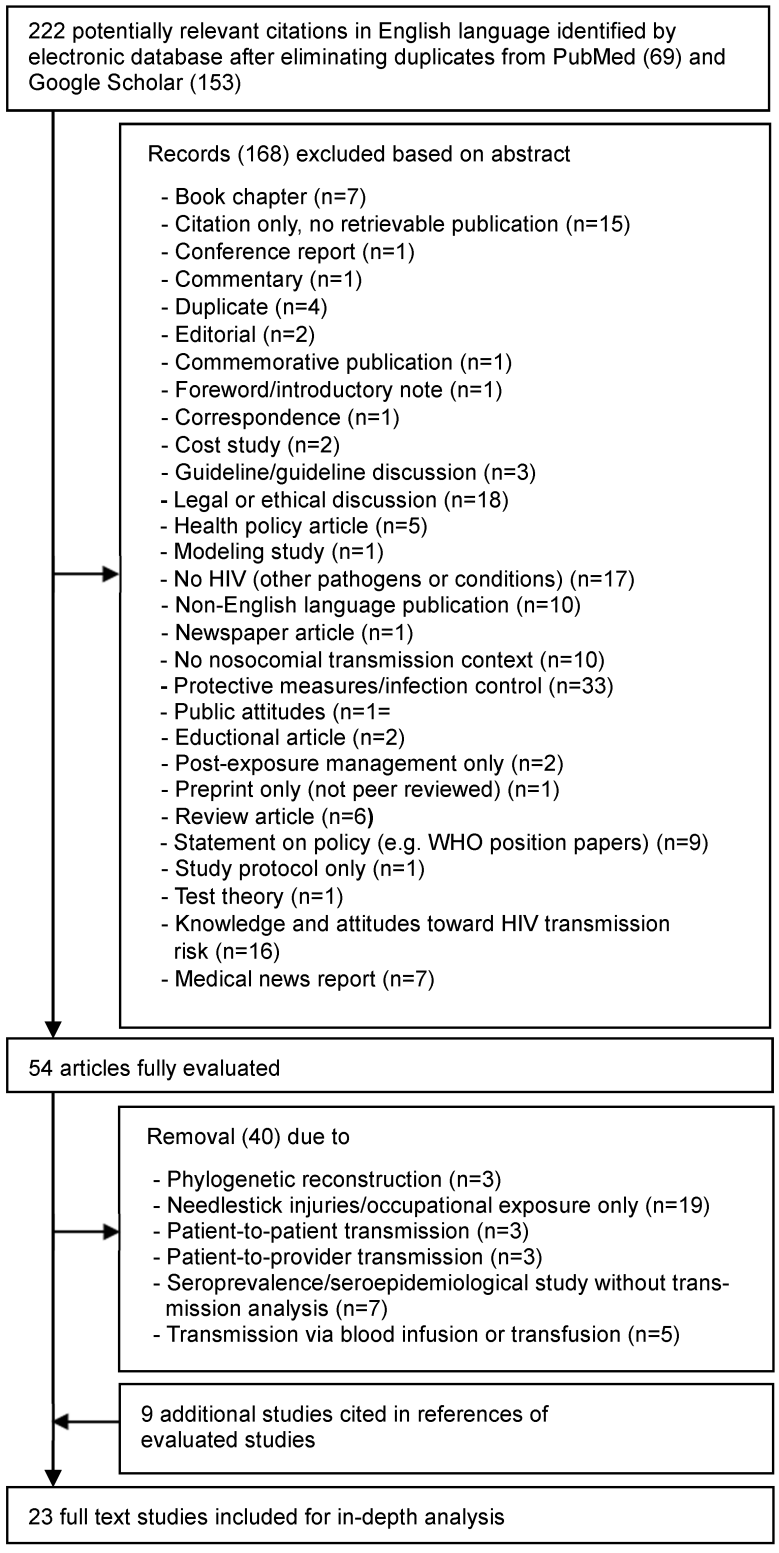
PRISMA flow diagram of study selection

Figure 1 (Fig. 1) shows the flow diagram of the literature search. In total, 222 abstracts were identified (69 in PubMed and 153 in Google Scholar). After exclusion of 168 records based on their abstracts, 54 full-text articles were reviewed. Of these, 14 studies met the eligibility criteria. An additional 9 studies, not captured by the search strategy, were identified through reference lists of full-text articles. In total, 23 studies, 7 peer-review transmission studies [7], [8], [13], [14], [15], [16], [17] addressing a total of 4 transmission events, and 16 look-back studies [9], [18], [19], [20], [21], [22], [23], [24], [25], [26], [27], [28], [29], [30], [31], [32], were included in the analysis.

### Study characteristics

The characteristics of the included studies are summarized in Table 1 (Tab. 1) and Table 2 (Tab. 2). All 23 studies originated from high-income countries and were published between 1987 and 2014. The majority were conducted in the United States (13/23; 56.5%), followed by France (4/23; 17.4%), the United Kingdom (3/23; 13.0%), Spain (2/23; 8.7%), and Israel (1/23; 4.3%). Notably, the most recent exposure period associated with a reported probable HCW-to-patient HIV transmission ended in March 2001, as described in the report by Mallolas et al. [16].

### Transmission studies

To date, seven publications have reported HIV transmission from HCWs to patients [7], [8], [13], [14], [15], [16], [17] (Table 1 (Tab. 1)). However, these reports describe only four distinct transmission events, as in three instances paired publications refer to the same patients. In two of these instances, one publication provides the epidemiological framework and risk estimates, while the companion article delivers molecular confirmation of the transmission event. In the third instance, the second publication represents a clarification or extension of the original report rather than a separate transmission event.

Across these four documented HCW-to-patient transmission events, the source HCW was a dentist in one event (the Florida dental cluster [7]), surgeons in two events (one orthopedic surgeon [13], [14] and one obstetrician [16], [17]), and a nurse in one atypical event [15]. Overall, these studies describe nine patients. Three cases were reported as isolated, probable transmissions, whereas the remaining cases occurred within a single confirmed outbreak—the Florida dental cluster.

Notably, specific percutaneous injuries or clearly defined exposure incidents were documented in two of the four events. In contrast, in the remaining two—including the confirmed patient cluster—no discrete procedural breach or identifiable transmission mechanism could be established despite extensive epidemiological and molecular investigation.

In this cluster, molecular epidemiology unequivocally linked an HIV-infected dentist to five infected patients. Although no single procedural injury or discrete exposure event could be identified, the genetic analyses demonstrated tight phylogenetic clustering and excluded alternative sources of infection [8]. Among 1,100 tested exposed individuals, this corresponds to an estimated transmission risk of 0.45% (5/1,100), with an exact two-sided 95% Clopper-Pearson CI of 0.15–1.06%. This interval corresponds to approximately one transmission per 676 to 95 treated patients.

In contrast, the single-case transmission investigations reported by Blanchard et al. [13] and Lot et al. [14], together included 983 tested exposed patients, yielded markedly lower point estimates. Under the assumption of one transmission event, the estimated transmission risks ranged from 0.003% to 0.566%, corresponding to an upper confidence bound of approximately one transmission per 177 procedures. Mallolas et al. [16] reported a probable HCW-to-patient HIV transmission during a caesarean section, supported by a documented percutaneous injury and strong phylogenetic relatedness between patient and obstetrician viruses but did no report any testing in additional patients.

### Look-back studies

In contrast to the small number of documented HCW-to-patient transmission events, sixteen look-back studies have systematically examined HIV infection rates among patients exposed to HIV-infected HCWs (Table 2 (Tab. 2)). Across these 16 investigations, a total of 32,899 patients were classified as potentially exposed, of whom 12,924 patients (39.3%) underwent HIV testing.

Across all identified HIV look-back studies, no HCW-to-patient transmission was observed. The studies covered a wide range of clinical settings and medical specialties and included between 41 and 2,310 tested patients per investigation (Table 2 (Tab. 2)). With the exception of the study by Astagneau et al. [30], all investigations involved exposure-prone procedures (EPPs). Follow-up completeness varied markedly, ranging from very limited testing of approximately 4% in the early investigation of Armstrong et al. [9] to near-complete ascertainment in the most recent cohort reported by Lam et al. [32]. Molecular epidemiological analyses were applied in three studies to exclude genetically related transmission events.

For the main analyses, in which zero transmissions were observed among tested patients (k=0), two-sided exact Clopper-Pearson 95% CIs were calculated to quantify the maximum transmission risk compatible with the observed data. The resulting upper confidence limits varied substantially across studies and ranged from 0.159% in the large cohort reported by Astagneau et al. [30] to 8.604% in the small study by Arnow et al. [25], depending on the number of patients tested. In the largest and more comprehensively investigated cohorts—Astagneau et al. [30], York et al. [21], von Reyn et al. [22], Longfield et al. [26], Jaffe et al. [28], and Donnelly et al. [29]—the upper 95% confidence limits in the main analyses were consistently below 0.32%, corresponding to fewer than one transmission per approximately 300 treated patients. 

To address uncertainty arising from incomplete follow-up and testing, a conservative sensitivity analysis was performed for each study by assuming that one HIV transmission had been missed and that this single event occurred anywhere within the entire exposed population, irrespective of testing status (k=1, n=all exposed, Scenario A). Under this deliberately pessimistic assumption, two-sided exact 95% Clopper-Pearson CIs were recalculated (Table 3 (Tab. 3)). Across all studies, the resulting upper confidence limits ranged from 0.074% in the study by Astagneau et al. [30] to 6.169% in the study by Arnow et al. [25], corresponding to a maximum plausible transmission risk of fewer than 1 in 1,360 exposed patients and 1 in 16 exposed patients, respectively.

Under this worst-case assumption of one undetected transmission event, none of the studies yielded an upper 95% confidence limit exceeding 6.2%, and the vast majority demonstrated upper limits below 0.5%, corresponding to fewer than one transmission per 200 exposed patients. These findings indicate that, even under deliberately pessimistic assumptions, the true HIV transmission risk from HCWs to patients remains tightly constrained to a very low range across all investigated settings.

When the hypothetical missed infection was restricted to untested exposed individuals only (Scenario B, Table 4 (Tab. 4)), the resulting upper confidence limits were driven almost exclusively by the size of the untested subgroup serving as the denominator. In studies with large numbers of untested exposed patients, such as Jaffe et al. [28] and von Reyn et al. [22], the assumption of a single missed infection was distributed over a large denominator, resulting in relatively low upper confidence limits that remained below approximately 0.1–0.5%, despite incomplete follow-up. In contrast, studies in which the majority of exposed patients were tested, leaving only very small untested subgroups, showed markedly inflated upper confidence limits under this deliberately pessimistic assumption. This effect was most pronounced in Danila et al. [20], Arnow et al. [25] and Lam et al. [32], where fewer than 50 untested individuals remained and the upper 95% confidence limits exceeded 10% or more. 

### Study quality and sources of bias in look-back investigations

Beyond the absolute number of tested patients and the resulting statistical uncertainty, look-back studies also differ substantially in their methodological quality, particularly with respect to case ascertainment, patient inclusion processes, and completeness of follow-up. These differences introduce important sources of bias that must be considered alongside CI estimates.

Armstrong et al. [9] conducted one of the earliest retrospective epidemiologic investigations of a general surgeon with advanced HIV infection. Patient follow-up was entirely passive: investigators were explicitly instructed not to directly contact the surgeon’s patients. This approach created substantial potential for selection bias, as HIV testing was voluntary and initiated solely at the patient’s request. Patients who presented for testing were therefore unlikely to be representative of the overall exposed cohort, severely limiting systematic outcome ascertainment.

In the dental practice investigation by Jaffe et al. [28], only a minority of potentially exposed patients underwent HIV testing, introducing substantial selection and response bias due to voluntary, self-initiated testing. Molecular sequence data were unavailable for 4 of the 28 known HIV-positive patients. Although epidemiological evidence suggested community acquisition for three of these individuals, the source of infection for one patient (Patient U) could not be determined with certainty, introducing residual uncertainty in outcome classification despite extensive molecular analyses in the remainder of the cohort. 

Porter et al. [18] employed a decentralized, general practitioner-led notification strategy, in which individual GPs—not public health authorities—were responsible for contacting patients and arranging testing. This design likely introduced significant selection bias, as testing depended on GP discretion, local practices, and patient willingness to participate, resulting in uneven and potentially non-representative follow-up.

In von Reyn’s study [22] participation was based on voluntary response following postal notification. Patients without a valid address (“undeliverables”, 22.9%) as well as non-responders (23.8%) were excluded before any opportunity for HIV testing. Even within the reachable cohort, the tested population was therefore selective, reflecting response bias; notably, 59 patients actively declined HIV testing.

In Astagneau´s look back study [30] selection bias arose because only patients with a valid postal address could be contacted; approximately 30% of potentially exposed patients were never informed and therefore excluded from any opportunity for testing. Response bias is present because HIV testing relied on voluntary return of serologic results following mailed notification; only 2,310 of 7,580 exposed patients (30.5%) provided test results, and tested patients differed slightly in sex distribution from the full exposed cohort.

In the study by Arnow et al. [25], selection bias was, to some extent, intentionally introduced. The investigation was explicitly designed to prioritize the confidentiality of the HIV-infected dentist. Patient notification was restricted to those who had undergone higher-risk procedures (categories III–V, broadly corresponding to exposure-prone procedures), and all communication was anonymized. While ethically justified, this strategy necessarily limited follow-up completeness and precluded comprehensive assessment of the full exposed population.

The investigation by Rogers et al. [23] illustrates a combination of multiple bias mechanisms. Selection bias occurred at the initial stage of cohort assembly, as a proportion of potentially exposed patients could not be contacted because no valid address was available and were therefore excluded before any opportunity for testing. This limitation was compounded by response bias, as a substantial proportion of contacted patients did not undergo systematic testing and some HIV test results were self-reported rather than laboratory verified. Additionally, the study could not identify patients for whom the surgeon acted as an assistant rather than the primary operator, leading to potential exposure misclassification and underascertainment of exposed individuals.

Similar limitations were present in the Israeli CDC look-back investigation [31]. Selection bias arose because only patients with available contact information could be notified and invited for testing, while unreachable patients were excluded from follow-up. Response bias further resulted from incomplete testing uptake among contacted patients, raising the possibility that tested individuals were not fully representative of all exposed patients.

In contrast, the study by York et al. [21] represents a methodological benchmark among look-back investigations. The researchers first identified all patients treated by three HIV-positive dentists during the period of presumed infectivity using a dental treatment database and then electronically linked these records to a central HIV registry containing mandatory test results for all active-duty military personnel. This approach effectively eliminated recall bias, minimized selection and response bias, and allowed near-complete outcome ascertainment without direct patient contact. The study thus set a methodological standard for subsequent look-back analyses.

## Discussion

Across more than four decades of published literature, only a single confirmed cluster of healthcare worker-to-patient HIV transmission has been documented worldwide, involving five patients treated by a dentist in Florida in the late 1980s. This cluster was conclusively established by detailed molecular epidemiological analyses demonstrating near-identical viral sequences and clear exclusion of alternative sources of infection [8]. Despite extensive investigation by the CDC [33], however, no specific procedural breach, accidental injury, or reproducible exposure pathway could be identified. Consequently, the Florida cluster is widely regarded as a singular, unexplained outlier rather than a representative model for occupational HIV transmission risk and cannot reasonably serve as a quantitative benchmark for contemporary risk estimation.

Beyond this unique event, only three isolated cases of probable HCW-to-patient HIV transmission have been reported, each supported by strong phylogenetic linkage. In the Florida dental cluster, five molecularly confirmed transmissions occurred among approximately 1,100 tested exposed patients, corresponding to an estimated transmission probability of 0.45% (95% exact Clopper-Pearson CI 0.15–1.06%), equivalent to roughly one transmission per 676 to 95 treated patients. The single-case investigations reported by Blanchard et al. [13] and Lot et al. [14] in which critical risk factors—including untreated HIV infection, high viral load, exposure-prone procedures, and documented percutaneous injury—coincided, yielded lower point estimates, with upper confidence bounds below 0.6% (approximately one transmission per 177 procedures). Two additional probable transmission events were described without systematic testing of other exposed patients, excluding meaningful estimation of transmission probability.

In a broader context, the widespread implementation of safety-engineered devices—such as those mandated under the U.S. Needlestick Safety and Prevention Act of 2000 [34] and the adoption of EU Council Directive 2010/32/EU [35] into national legislation across European countries—has likely contributed to a sustained reduction in needlestick injuries [36], [37] and, therefore, to a decline in occupationally acquired HIV infections among HCWs. By lowering the prevalence of HIV infection within the healthcare workforce, in addition to the preventive effect of ART, these measures may have reduced the pool of potentially infectious HCWs, thereby secondarily decreasing the probability of HCW-to-patient transmission at the population level.

These transmission reports, although rare, stand in marked contrast to the consistently negative findings of the 16 large look-back studies included in this review, encompassing many tens of thousands of patients treated by HIV-infected HCWs. At first glance, this apparent absence of transmission might suggest an exceedingly low or negligible nosocomial risk. However, the absence of observed events alone is insufficient to quantify risk. Because all look-back studies reported zero transmissions, their interpretation depends not on point estimates but on statistical power, as reflected by the width of their CIs. Studies with small numbers of tested patients cannot exclude non-trivial transmission risks, whereas larger cohorts permit increasingly narrow upper bounds on the plausible risk.

Indeed, as indicated by the upper limits of the exact 95% CIs, even the largest and most comprehensive investigations remain statistically compatible with residual transmission risks of up to approximately one transmission per 300 treated patients. This uncertainty is further amplified by methodological limitations inherent to many look-back investigations. At least eight of the 16 included studies were affected by substantial sources of bias, including severe selection and response bias, exposure misclassification, and incomplete exclusion of alternative routes of HIV acquisition.

Sensitivity analyses were therefore required not merely to address incomplete follow-up, but to account for these underlying methodological constraints. Two complementary worst-case sensitivity analyses were performed. Distributing a hypothetical missed infection across the entire exposed cohort (Scenario A) yields conservative population-level upper bounds, whereas restricting the event exclusively to untested exposed individuals produces higher—and in some studies extreme—confidence limits. When assuming a single undetected HCW-to-patient HIV transmission, the resulting estimates demonstrate that non-zero transmission risks remain statistically compatible with the available data even under deliberately conservative assumptions. Although in the vast majority of studies the resulting upper 95% confidence limits remained below 0.5%, this still corresponds, in the most unfavorable case, to a residual risk of up to one transmission per 200 exposed patients.

Under the alternative Scenario B restricting a hypothetical missed transmission to untested exposed individuals, upper 95% confidence limits spanned a very wide range—from approximately 0.11% (≈1 in 950) to 41.3% (≈1 in 2.4). Stratification by the size of the untested subgroup shows that these extreme values are driven by small denominators rather than by evidence of increased biological transmissibility. Where several hundred or more individuals remained untested, upper confidence limits remained below approximately 1%, whereas very small untested subgroups produced disproportionately inflated estimates.

Taken together, these findings indicate that the available empirical data are insufficient to exclude low-level transmission risk on statistical and methodological grounds alone. This residual uncertainty underscores the need for clear, prospective, viral-load-based guidelines for the management of HCWs living with HIV, rather than reliance on retrospective zero-event observations. Contemporary policy recommendations, such as those issued by the Society for Healthcare Epidemiology of America (SHEA) [38] and the United Kingdom Advisory Panel for Healthcare Workers Infected with Bloodborne Viruses (UKAP) [39], are not based on assertions of zero risk but on a regulatory assessment of robust biological evidence indicating that sustained viral suppression effectively eliminates infectiousness (“undetectable equals untransmittable”, U=U). Accordingly, these guidelines permit HCWs living with HIV who maintain durable virological suppression to continue clinical practice without restriction, including the performance of EPPs, thereby replacing earlier infection-status-based exclusions with a viral-load-driven risk assessment.

By explicitly acknowledging uncertainty and applying conservative sensitivity analyses, this review avoids any impression of false reassurance. At the same time, it demonstrates that even under deliberately pessimistic assumptions, the estimated transmission risks remain incompatible with routine or systematic HCW-to-patient HIV transmission. In this sense, U=U-based guidelines are not weakened by the limitations of look-back studies; rather, they are supported by a transparent appraisal of uncertainty and by the consistent finding that all plausible risk estimates remain very low. Such guidelines are essential both to ensure patient safety through virological suppression and to protect the professional rights and autonomy of HCWs living with HIV.

Finally, although contemporary guidelines are primarily grounded in biological evidence, the historical body of look-back studies appears to have played an important contextual and enabling role. Together with the very small number of documented HCW-to-patient transmission events, these investigations demonstrated that occupational HIV transmission is rare—even in the pre-antiretroviral therapy era, during exposure-prone procedures, and under conditions of incomplete follow-up. By consistently failing to identify frequent or widespread HCW-to-patient HIV transmission, look-back studies helped to allay early fears of uncontrolled occupational spread [40] and can be viewed as a necessary precondition that enabled the subsequent shift from a binary, infection-based exclusion model toward a risk-based, viral-load-driven framework.

## Notes

### Author’s ORCIDs

- Diel R: https://orcid.org/0000-0001-8304-7709
- Gottschalk R: https://orcid.org/0000-0003-0422-6456
- Nienhaus A: https://orcid.org/0000-0003-1881-7302

### Competing interests

The authors declare that they have no competing interests.

## Supplementary Material

Search strategies

## Figures and Tables

**Table 1:**
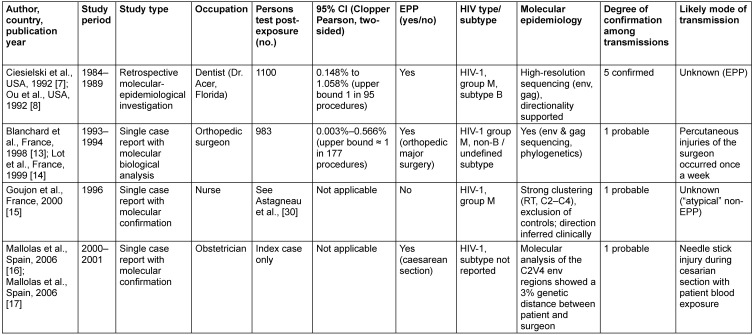
Results of suggested direct HIV transmission by HCW

**Table 2:**
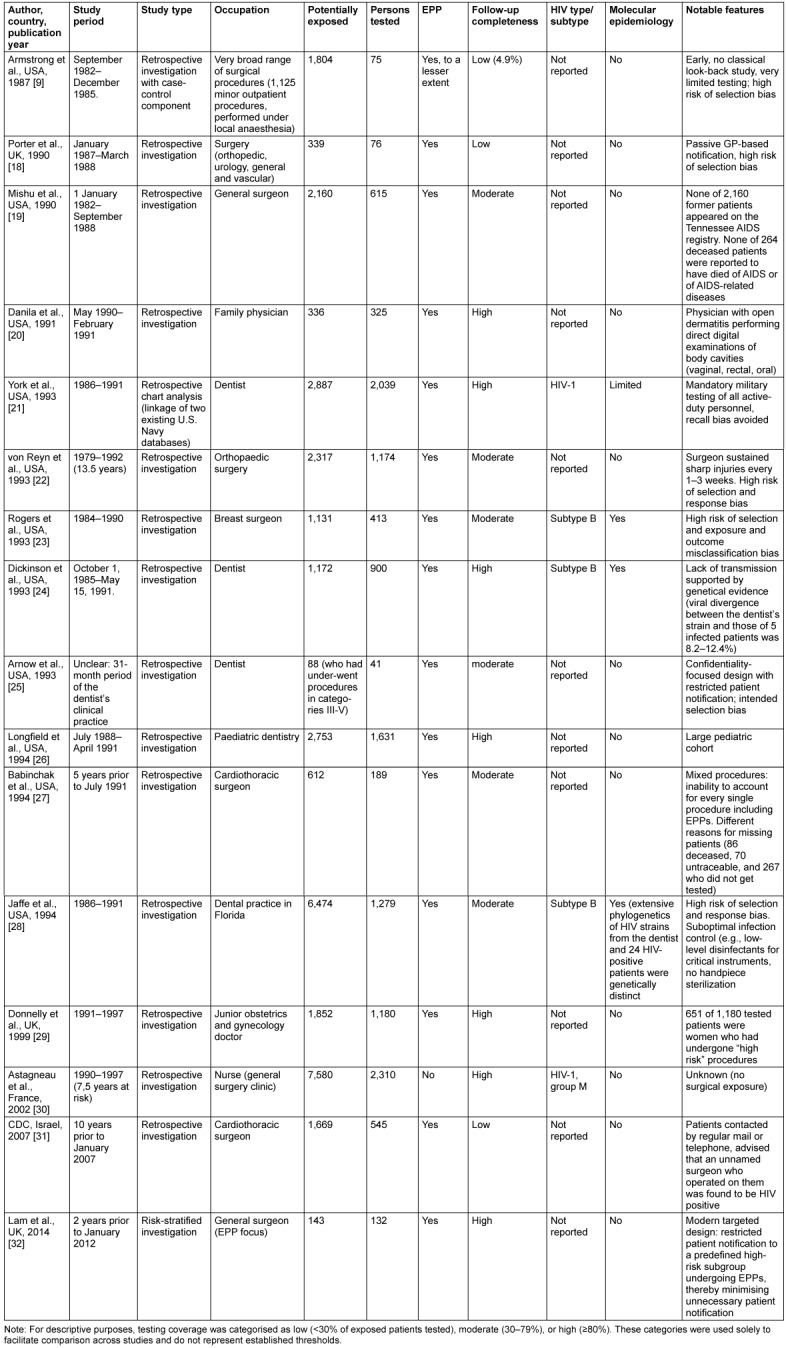
Characteristics of included HIV look-back studies

**Table 3:**
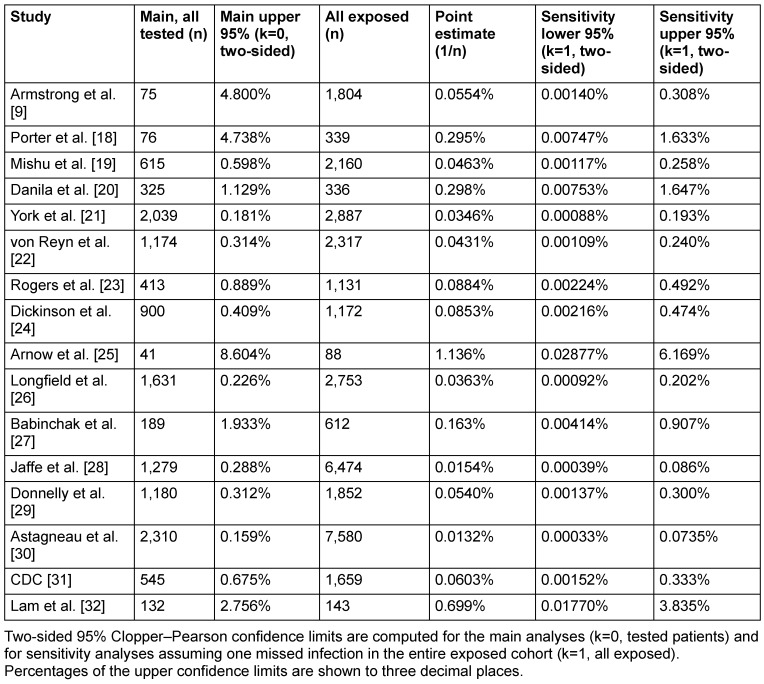
Main and sensitivity analysis (Scenario A)

**Table 4:**
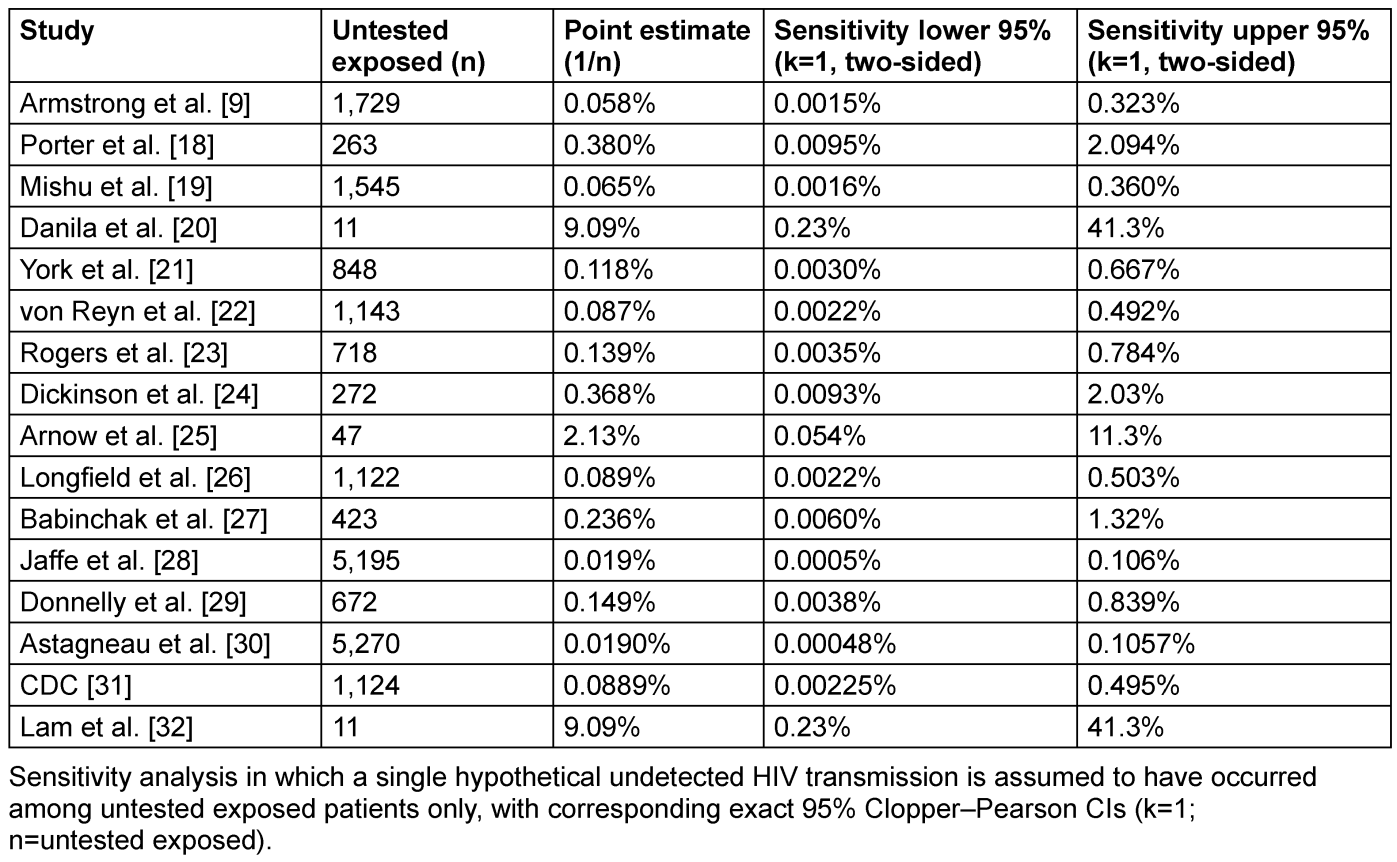
Alternative sensitivity analysis (scenario B)
