# Supplementary material for: Risk of HIV transmission by healthcare workers – a systematic review
Source: GMS Hyg Infect Control. 2026 Mar 13;21:Doc32. doi: 10.3205/dgkh000641 (PMC13103719; doi:10.3205/dgkh000641)
Supplement: Search strategies [file HIC-21-32-s-001.pdf]

Attachment 1

Search strategies

PubMed

The PubMed search strategy was developed to identify original reports, outbreak investigations, and evaluation studies on HIV transmission from HCWs to patients. It was intentionally designed to maximize specificity for provider-to-patient transmission while excluding the far more prevalent literature on occupational exposure, patient-to-provider transmission, and secondary reviews.

("HIV"[MeSH Terms] OR "HIV"[All Fields])
AND
("Health Personnel"[MeSH Terms] OR "healthcare worker"[All Fields] OR "health care worker"[All Fields] OR "medical staff"[All Fields] OR "health personnel"[All Fields])
AND
("Disease Transmission, Infectious"[MeSH Terms] OR "Cross Infection"[MeSH Terms] OR "Iatrogenic Disease"[MeSH Terms]
 OR (infectious[All Fields] AND transmission[All Fields])
 OR (HIV[All Fields] AND transmission[All Fields]))
AND
("provider to patient"[All Fields] OR "HCW to patient"[All Fields]
 OR "iatrogenic transmission"[All Fields]
 OR "healthcare-associated"[All Fields]
 OR (transmission[All Fields] AND surgery[All Fields]))
NOT
("needle-stick"[All Fields] OR "occupational exposure"[All Fields]
 OR "patient-to-healthcare worker"[All Fields] OR "patient to provider"[All Fields])
NOT
(review[pt] OR systematic[sb])
AND
("Case Reports"[pt] OR outbreak[All Fields] OR "Evaluation Study"[pt])

Google Scholar

Because Google Scholar does not support controlled vocabulary, field tags, or publication-type filters, a highly specific keyword-based strategy with multiple exclusion terms was used. This approach was intended to suppress the dominant literature on occupational exposure and patient-to-provider transmission and to enrich retrieval of original reports on provider-to-patient HIV transmission.

"HIV transmission" AND
("healthcare worker to patient" OR "health care worker to patient"
 OR "provider to patient" OR "surgeon to patient"
 OR "iatrogenic HIV" OR "nosocomial HIV")
-"patient to healthcare worker"
-"needle stick"
-"occupational exposure"
-"review"
-"literature review"
-"case report"
-"exposition"
-"exposition accidentell"

Attachment 1 to: Diel R, Gottschalk R, Nienhaus A. Risk of HIV transmission by healthcare workers – a systematic review. GMS Hyg Infect Control. 2026;21:Doc32. DOI: 10.3205/dgkh000341
